# Transcriptome Analysis Reveals Differences in Molecular Mechanisms Between Salt-Tolerant and Salt-Sensitive Rice (*Oryza sativa* L.) Varieties Under Salt Stress

**DOI:** 10.3390/cimb47100832

**Published:** 2025-10-10

**Authors:** Yu Han, Chenyang Wu, Xue Ji, Mengran Yang, Hongyu Zhu, Zhongyou Pei, Mingnan Qu, Lijun Qu, Zhibin Li, Shuangyong Yan

**Affiliations:** 1 Department of Agriculture and Resources and Environment, Tianjin Agricultural University, Tianjin 300384, China; hy18812778669@163.com (Y.H.);; 2 Institute of Crop Research, Tianjin Academy of Agricultural Sciences, Tianjin 300381, China; 3 Jiangsu Key Laboratory of Crop Cu ltivation and Physiology, College of Agricultural, Yangzhou University, Yangzhou 225009, China; 4 Enterprises Key Laboratory of Hybrid Japonica Rice, Tianjin 300457, China

**Keywords:** rice (*Oryza sativa* L.), salt stress, transcriptomic analysis, salt tolerance mechanism

## Abstract

To elucidate the molecular mechanisms underlying salt tolerance in rice (*Oryza sativa* L.), this study investigated differential transcriptional responses during the tillering stage. Salt-tolerant (N14) and salt-sensitive (N6) varieties were subjected to 0.3% and 0.6% NaCl treatments for 72 h, and their transcriptomes were analyzed via RNA-Seq. The results revealed distinct response strategies: 372 differentially expressed genes (DEGs) were identified in N14 and 393 in N6, with only 17 genes responding similarly. Gene Ontology (GO) analysis showed the tolerant N14 activated protein phosphorylation and lipid transport, primarily in the membrane and extracellular regions (e.g., ATP binding), whereas the sensitive N6 activated photosynthesis and protein folding, localized to chloroplasts and peroxisomes. KEGG analysis highlighted the activation of “Plant-pathogen interaction” in N14 versus “Metabolic pathways” in N6. Differential transcription factor activation was also observed, with N14 mobilizing 52 TFs (mainly WRKY and MYB) and N6 mobilizing 36 TFs (mainly MYB and b-ZIP). This study demonstrates that N14 and N6 utilize significantly different molecular pathways to cope with salinity, providing a crucial theoretical foundation for identifying novel salt tolerance genes and developing molecular breeding strategies.

## 1. Introduction

Soil salinization is an environmental factor that severely affects crop growth [1]. Under saline–alkali conditions, plants regulate specific molecular mechanisms to maintain their normal growth and development. Indeed, extensive molecular research has been conducted on these mechanisms, utilizing proteomics and comparative transcriptomics [2]. Li et al. (2017) conducted a comparative transcriptomic study on salt-tolerant and salt-sensitive rice genotypes, finding that the tolerant variety (Dongdao-4) relied primarily on a greater capacity for osmotic adjustment (accumulation of proline and soluble sugars) and a more efficient ROS-detoxifying system (such as CAT activity) to mitigate stress, a mechanism independent of ion toxicity [3]. Nevertheless, given the extreme complexity of tolerance mechanisms and the diversity of strategies among different rice varieties, comparative analyses of additional genotypes with different genetic backgrounds remain crucial for revealing specific regulatory networks and key genes. The plant salt tolerance mechanism is highly complex, encompassing multiple aspects such as ion homeostasis, ion compartmentalization, biosynthesis of Osmo protectants, and antioxidant defense. Salt stress not only causes direct osmotic toxicity but also has indirect oxidative stress effects [4]. Employing modern molecular biological techniques can accelerate the breeding process of salt-tolerant rice and offer new avenues for speeding up the genetic improvement of rice salt tolerance [5]. Rice exhibits varying sensitivity to salinity throughout its life cycle; the seedling stage and the tillering stage, in particular, are considered “extremely sensitive” periods and key stages for evaluating salt tolerance. Research focusing on these sensitive phases has identified that the tiller number is the trait most severely inhibited by salinity, making it a primary criterion for salt tolerance identification [6]. Furthermore, tolerance mechanisms are highly specific to the stressor. Crucially, studies have demonstrated that tolerance to neutral salt (NaCl) stress and tolerance to alkaline stress are not significantly correlated in rice. This finding underscores that tolerance to one stress type does not imply tolerance to the other. Therefore, it is necessary to conduct distinct molecular investigations focused specifically on NaCl stress during the critical productive stage (the tillering stage) [7]. The salt tolerance of rice is highly complex, being a typical quantitative trait controlled by multiple genes and greatly influenced by the environment, with a large range of heritability [8]. The reported loci mainly include qSKC-1 and Saltol, which are located on rice chromosome 1 [9,10,11]. The salt-tolerant QTL qSTK1.1 identified from Seawater Rice 86 is also located in the Saltol region and encodes the same amino acid sequence as SKC1 (OsHTK1;5) from Nona Bokra [12]. Under specific physiological conditions, the sum of the transcriptional products within a cell constitutes the transcriptome. These transcriptional products encompass a variety of types, including mRNA, tRNA, rRNA, snRNA, and so on. From a narrow perspective, however, the transcriptome refers to the entirety of all mRNAs within a cell. When rice is subjected to salt stress, differentially expressed genes in different tissues or at different developmental stages can be identified [13]. Therefore, transcriptome sequencing technology is a more precise method for measurement at the transcriptional level. Fang et al. [14] compared the transcriptomes and revealed that the expression of two sucrose phosphate synthase (SPS) genes (LOC_Os01g69030 and LOC_Os08g20660) and two glutathione S-transferase (GST) genes (LOC_Os06g12290 and LOC_Os10g38740) was upregulated in salt-tolerant and salt-sensitive varieties. Meanwhile, the expression of LOC_Os09g12660 (a glucose-1-phosphate acetyltransferase gene) and two GST genes was also upregulated. A candidate gene that mediates salt tolerance, OsRCI2-8 (Os06g0184800), was identified through a combination of linkage mapping and transcriptome profiling analysis [15]. A comparative transcriptomic analysis was conducted between two rice genotypes, namely the salt-sensitive variety Chao 2R and the salt-tolerant variety RPY geng (japonica), under salt stress conditions. The results revealed that the expression of genes involved in normal life processes in Chao 2R was significantly disrupted by salt stress. In contrast, RPY geng (japonica) was able to modulate the expression of numerous stress-responsive genes to cope with the same level of salt stress, including those associated with secondary metabolism and oxidation–reduction processes [16]. Collectively, the research demonstrates the significance of RNA sequencing technology in pinpointing key salt-tolerant metabolic pathways and uncovering salt-tolerant candidate genes within plants.

Therefore, this study utilized the salt-sensitive N6 and salt-tolerant N14 varieties to investigate differential gene expression and regulatory mechanisms under salt stress using RNA-Seq, aiming to provide a theoretical basis for the genetic improvement of rice salt tolerance.

## 2. Materials and Methods

### 2.1. Source of Test Materials

To screen for salt-tolerant rice germplasm, 400 rice materials were evaluated for salt tolerance. Plump and uniformly sized seeds were selected, surface-sterilized, placed in germination boxes, and completely submerged in sterile distilled water. The germination boxes were placed in a 30 °C constant temperature incubator, first undergoing 24 h of imbibition in complete darkness. Upon germination, the culture conditions were immediately switched to a photoperiod of 12 h dark/12 h light, and cultivation continued for 1 week to reach the seedling stage. Subsequently, the 1-week-old seedlings were subjected to stress treatment using a series of NaCl concentration gradients: 0% (CK), 0.2% (T1), 0.4% (T2), and 0.6% (T3). After 21 consecutive days of treatment, the relative salt injury rate for each material was measured and calculated. Based on these identification results, the extremely salt-tolerant material N14 and the salt-sensitive material N6 were ultimately selected for subsequent research.

### 2.2. Experimental Methods

Using salt-sensitive rice variety N6 and salt-tolerant rice variety N14 as experimental materials, pot experiments were conducted. After the rice plants reached the tillering stage, a control group and two salt concentration levels (0.3% NaCl and 0.6% NaCl) were established to induce salt stress. After 72 h of stress, leaves were collected and rapidly frozen with liquid nitrogen. Subsequently, RNA-Seq technology was applied to compare the differences in transcriptional responses to salt stress between the two varieties and to investigate the metabolic pathways and differentially expressed genes in response to salt stress under different levels of resistance.

#### 2.2.1. Transcriptome Sequencing

Transcriptome sequencing was performed by Shanghai Personal Biotechnology Co., Ltd. (Shanghai, China). Briefly, after the samples passed quality control, mRNA was enriched using magnetic beads with Oligo(dT) and then fragmented. The fragmented mRNA was used as a template to synthesize double-stranded cDNA. The purified cDNA underwent end repair, 3′ end addition of ‘A’ bases, and sequencing adapter ligation, followed by PCR amplification to obtain the cDNA library. After the library passed quality control, it was sequenced on an Illumina sequencing platform.

#### 2.2.2. Differential Expression Analysis and Functional Enrichment

Differential expression analysis between the two comparison groups was performed using DESeq (v1.38.3) software. DESeq was used to analyse gene expression differences, with the screening criteria for differentially expressed genes being a fold change of |log2FoldChange| > 1 and a significance *p* < 0.05.

GO enrichment analysis was performed using topGO (v2.50.0). The hypergeometric distribution method (significance threshold: *p* < 0.05) was used to calculate *p*-values and identify GO terms significantly enriched in differentially expressed genes (all/up/down), thereby determining the primary biological functions of differentially expressed genes. KEGG pathway enrichment analysis was performed using Cluster Profiler (v4.6.0) software, focusing on significantly enriched pathways with *p*-values < 0.05. Gene set enrichment analysis was performed using GSEA (v4.1.0) software, followed by statistical testing to determine whether predefined gene sets were enriched at the top or bottom of the ranking table.

### 2.3. Real-Time Quantitative PCR

Total RNA was reverse transcribed into first-strand cDNA using the Uni All-in-One First-Strand cDNA Synthesis SuperMix for qPCR (One-Step gDNA Removal) (TransScript) kit (TransGen Biotech, Beijing, China). The resulting cDNA stock solutions (original concentration approx. 1300 ng/μL) were diluted with nuclease-free water and standardized to a uniform working concentration of 65 ng/μL. Subsequent qRT-PCR reactions were performed on an ABI 7500 Fluorescence Quantitative PCR Instrument (Applied Biosystems, Foster City, CA, USA). The total volume of each reaction mixture was 20 μL, with the specific formulation detailed in Table 1. The PCR cycling conditions are listed in Table 2. Finally, relative gene expression levels were calculated using the 2 ^−ΔΔCt^ method.

### 2.4. Data Visualization Software

Data visualization for this study was performed as follows: Enrichment analysis bubble plots were generated using the Weishengxin (https://www.bioinformatics.com.cn, accessed on 27 August 2025) online platform. The Venn diagram was drawn using the Venny 2.1 online tool (http://bioinfogp.cnb.csic.es/tools/venny/, accessed on 27 August 2025). The hierarchical clustering heatmap, the transcription factor distribution bar chart, and the qRT-PCR validation graphs were all plotted using Origin Pro 2024 software. The summary schematic model was constructed using BioRender.com(https://BioRender.com, accessed on 27 August 2025) software. All figures were finally assembled and typeset using Adobe Illustrator (2024).

## 3. Results

### 3.1. Differences in Plant Phenotypes of Different Salt Stress-Tolerant Varieties

**Figure 1:**
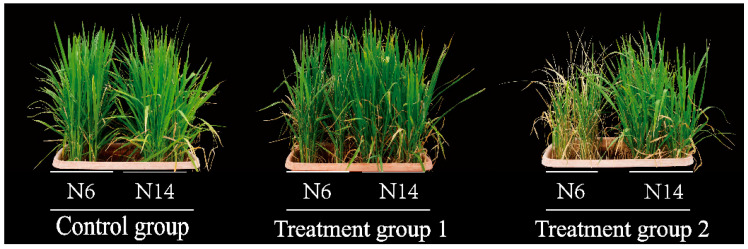
Phenotypic changes in rice plants after salt stress at different concentrations. Treatment group 1 means 0.3% NaCl, Treatment group 2 means 0.6% NaCl.

The two varieties tested in this study showed significant differences in salt tolerance. Under 0.3% NaCl treatment, the leaves of N6 and N14 darkened in colour compared to the control group. N6 had more yellowing leaves than the control group, but the number of yellowing leaves in N14 did not change significantly compared to its respective control group. Under 0.6% NaCl treatment, N6 had significantly more yellowing leaves than N14, and the leaf colour lightened (Figure 1).

### 3.2. Transcriptome Sequencing of Salt-Tolerant and Salt-Sensitive Varieties

Transcriptome sequencing was performed on samples and corresponding controls of the two varieties under two salt stress conditions (0.3% and 0.6%), yielding transcriptome sequencing data from a total of 18 samples. A total of 133.67 Gb of clean data was obtained, with each sample yielding more than 5.88 Gb of clean data. The Q30 content was above 94.28% (Table 3). Each sample was aligned with reads that had only one best-matching position on the reference genome, with an alignment rate of 97.58–98.83%. This indicates that the overall sequencing quality was high, and the data can be used for subsequent bioinformatics analysis.

### 3.3. Analysis of Differentially Expressed Genes Among Different Varieties Under Salt Stress Conditions

Differentially expressed genes (DEGs) of N6 and N14 under salt stress. The distribution of DEGs under different treatments is shown in the figure.

**Figure 2:**
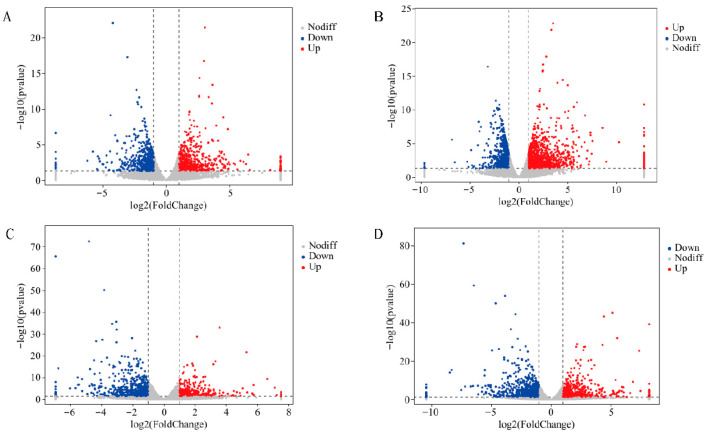
Volcano plot of differentially expressed genes between N6 and N14. Red indicates significantly upregulated genes, blue indicates significantly downregulated genes, and grey indicates genes with no significant difference. (**A**) Differentially expressed genes in N6 under 0.3% salt stress. (**B**) Differentially expressed genes in N6 under 0.6% salt stress. (**C**) Differentially expressed genes in N14 under 0.3% salt stress. (**D**) Differentially expressed genes in N14 under 0.6% salt stress.

Under 0.3% salt stress conditions, the salt-sensitive variety N6 exhibited a total of 1189 differentially expressed genes (DEGs), with 639 DEGs showing upregulation and 550 downregulation (Figure 2A). Under 0.6% salt stress conditions, a total of 1997 DEGs were identified, with 1226 showing upregulation and 771 downregulation (Figure 2B). The salt-tolerant variety N14 exhibited 1043 differentially expressed genes under 0.3% salt stress conditions, with 431 DEGs showing upregulation and 612 showing downregulation (Figure 2C); under 0.6% salt stress conditions, there were 1299 differentially expressed genes, with 599 DEGs showing upregulation and 700 showing downregulation (Figure 2D). This indicates that different salt stress concentrations and variety differences lead to distinct salt stress response mechanisms.

To reveal more general transcriptional differences in salt-sensitive and salt-tolerant varieties in response to salt stress, we further analyzed differentially expressed genes. The results showed that in the salt-sensitive variety N6, 252 genes were upregulated and 190 genes were downregulated under both salt concentration treatments; in salt-tolerant materials, 167 genes were upregulated and 354 genes were downregulated (Table 4) (Supplementary S1).

**Figure 3:**
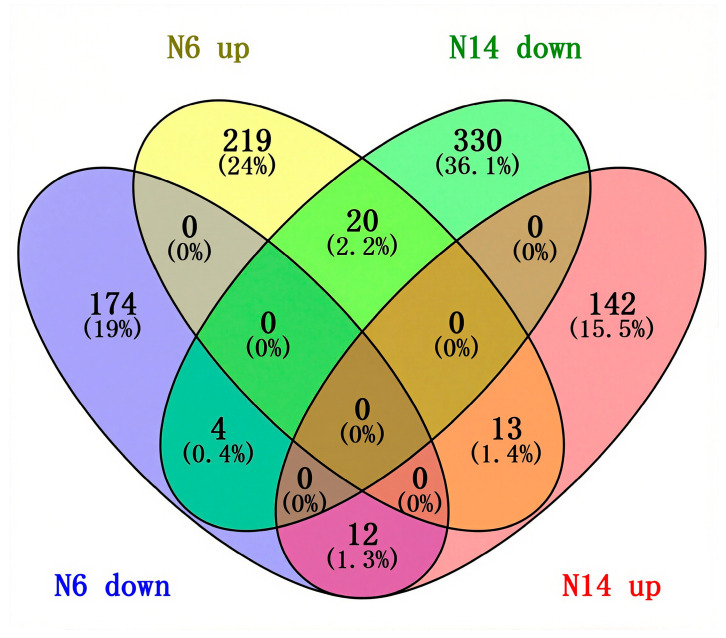
Venn diagram of commonly differentially expressed genes.

The results of the Venn diagram analysis of co-differentially expressed genes showed that there were a total of 49 genes that were differentially expressed in both N6 and N14. Among these, 4 genes were downregulated in both materials, 13 genes were upregulated in both materials, 12 differentially expressed genes were downregulated in N6 and upregulated in N14, and 20 differentially expressed genes were upregulated in N6 and downregulated in N14. This indicates that salt-sensitive and salt-tolerant varieties share common salt response genes, but the majority of genes exhibit opposite directions of differenial expression, which may be an important reason for the differences in salt tolerance between varieties. In the salt-sensitive variety N6, there were 219 differentially expressed genes that were specifically up-regulated and 174 that were specifically down-regulated. In the N14 variety, there were 142 differentially expressed genes that were specifically up-regulated and 330 that were specifically down-regulated. Therefore, there are significant differences in the response to salt stress between the salt-tolerant rice variety N14 and the salt-sensitive rice variety N6 (Figure 3).

### 3.4. Analysis of Differentially Expressed Genes in Salt-Tolerant and Salt-Sensitive Varieties in Response to Salt Stress

**Figure 4:**
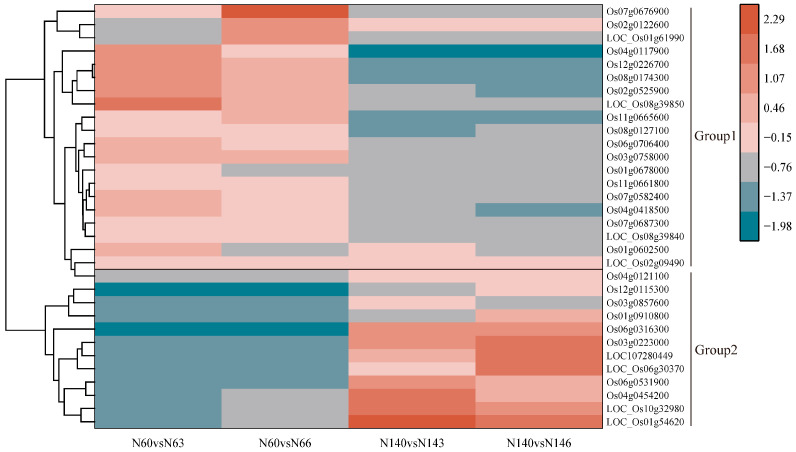
Common differentially expressed genes with different regulatory directions.

Using heatmaps, we performed cluster analysis on 32 differentially expressed genes with opposite regulatory directions in N6 and N14, and classified the cluster results into two groups (Group 1 and Group 2) (Figure 4) (Supplementary S2). The results showed that the expression levels of genes such as Os03g0758000, Os04g0117900, LOC_Os08g09490, and other genes showed significantly upregulated expression levels under salt stress in N6, but were significantly downregulated in N14. In contrast, LOC_Os01g54620, LOC_Os10g32980, Os04g0454200, and other genes were significantly downregulated in N6 under salt stress but significantly upregulated in N14, indicating that some genes in N6 and N14 exhibit opposite regulatory directions upon salt stress. This suggests that there are indeed significant differences in the response to salt stress between N6 and N14. Additionally, LOC_Os06g45140, LOC107278492, Os02g0575700, and Os10g0550900 were downregulated genes in both N6 and N14.Genes such as Os12g0600100, LOC_Os05g44340, and Os05g0247100 were all upregulated genes.

### 3.5. Analysis of Genes Specifically Upregulated in Salt-Tolerant Varieties

**Figure 5:**
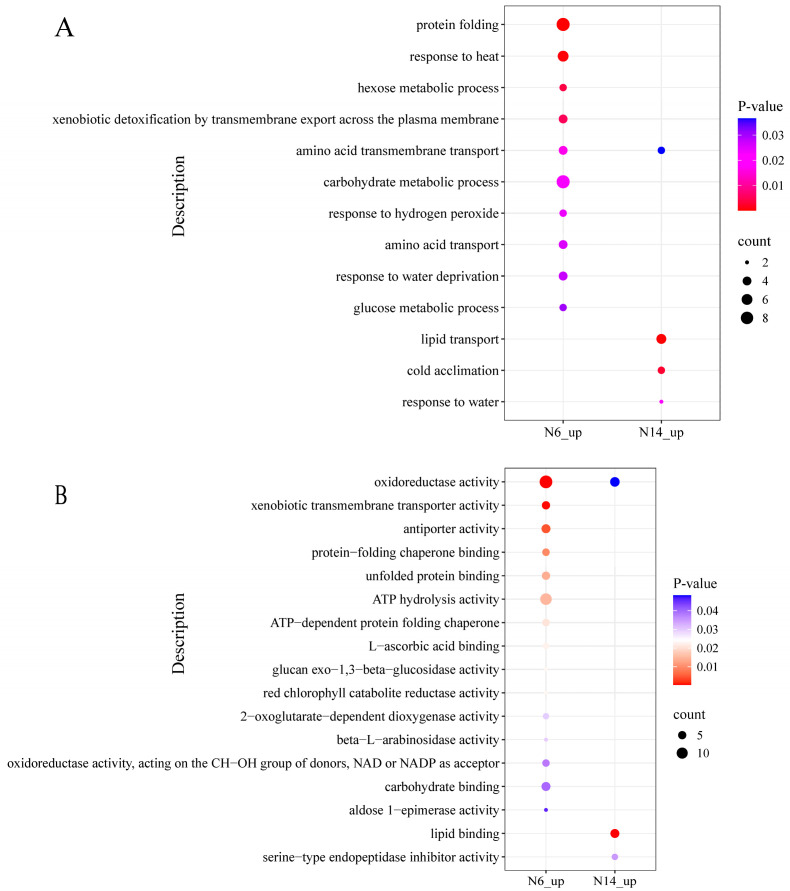
GO enrichment analysis of differentially expressed genes. (**A**) Biological processes in which both N6 and N14 are upregulated. (**B**) Molecular functions in which both N6 and N14 are upregulated.

N14 exhibits 142 uniquely upregulated differentially expressed genes under salt stress. GO enrichment analysis revealed (Figure 5A) that the differentially expressed genes in the salt-tolerant variety N14 were primarily enriched in biological processes such as lipid transport, cold acclimation, and response to water; whereas in the salt-sensitive variety N6, the uniquely upregulated genes were primarily enriched in biological processes such as protein folding and response to heat. In the biological process of amino acid transmembrane transport, both salt-tolerant and salt-sensitive varieties showed upregulation.

At the cellular component level, differentially expressed genes in the salt-tolerant variety showed an upregulation trend in the extracellular region, while differentially expressed genes in the salt-sensitive variety showed upregulation in peroxisomes (Supplementary S3). At the molecular function level (Figure 5B), differentially expressed genes in salt-tolerant varieties showed significant upregulation in lipid binding and serine-type endopeptidase activity. However, in the molecular function process of oxidoreductase activity, both salt-tolerant and salt-sensitive varieties showed gene expression without significant differences in expression patterns.

In N14, KEGG enrichment analysis indicated that the upregulated differentially expressed genes were mainly involved in metabolic pathways and the biosynthesis of secondary metabolites, suggesting that salt stress may have promoted the synthesis of secondary metabolites and overall metabolic activity in N14. In N6, the upregulated differentially expressed genes were primarily involved in metabolic pathways and the biosynthesis of secondary metabolites, suggesting that salt stress may have activated the secondary metabolic defence mechanisms in N6. These results reveal the metabolic regulatory mechanisms of rice under different treatment conditions, providing important clues for understanding its stress responses (Supplementary S3).

### 3.6. Analysis of Genes Specifically Downregulated in Salt-Tolerant Varieties

**Figure 6:**
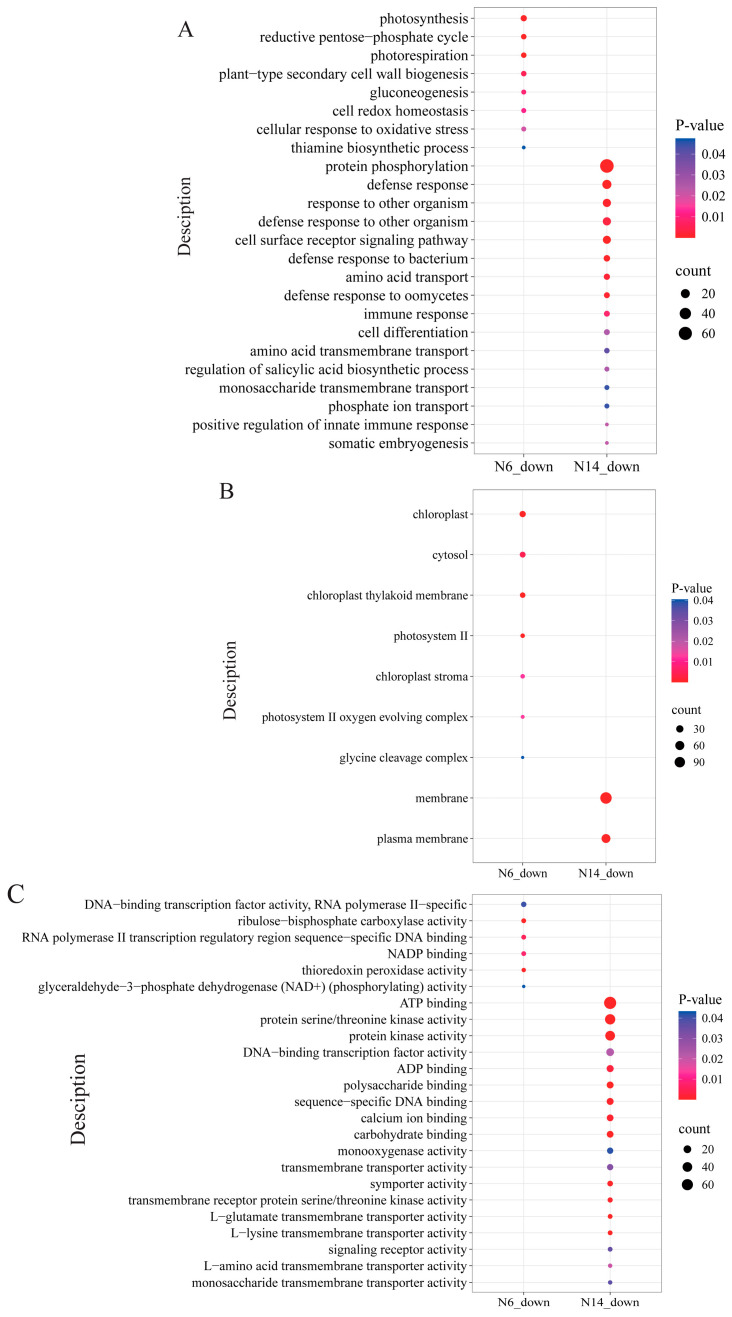
GO enrichment analysis of differentially expressed genes. (**A**) Biological processes in which both N6 and N14 are downregulated. (**B**) Cellular components in which both N6 and N14 are downregulated. (**C**) Molecular functions in which both N6 and N14 are downregulated.

When analysing the differentially expressed genes between salt-tolerant and salt-sensitive varieties, it was found that, in terms of biological processes (Figure 6A), the differentially expressed genes in salt-tolerant varieties were primarily concentrated in processes such as protein phosphorylation, defence response, and response to other organisms, exhibiting significant downregulation. At the cellular component level (Figure 6B), differentially expressed genes in salt-tolerant varieties showed a downregulated expression trend in the membrane and plasma membrane components, while differentially expressed genes in salt-sensitive varieties exhibited downregulated expression in components such as chloroplast, cytosol, and chloroplast thylakoid membrane. At the molecular function level (Figure 6C), differentially expressed genes in salt-tolerant varieties showed significant downregulation in functions such as ATP binding, protein serine/threonine kinase activity, and protein kinase activity, while the differentially expressed genes in salt-sensitive varieties showed significant downregulation in functions such as NADP binding, thioredoxin peroxidase activity, and ribulose-bisphosphate carboxylase activity. Interestingly, during the downregulation process, there were no identical biological processes, cellular components, or molecular functions between salt-tolerant and salt-sensitive varieties.

In N6, KEGG enrichment analysis indicated that downregulated differentially expressed genes were primarily enriched in key metabolic processes such as metabolic pathways, carbon fixation in the Calvin cycle, and photosynthesis, suggesting that salt stress significantly inhibited photosynthesis and carbon metabolism in rice in N6. In N14, downregulated differentially expressed genes were primarily concentrated in plant-pathogen interactions and diterpenoid biosynthesis, suggesting that salt stress may have enhanced N14’s defence mechanisms (Supplementary S3).

Overall, the common differentially expressed genes between N6 and N14 were primarily enriched in upregulation processes in N6, while in N14, they were more enriched in downregulation processes.

### 3.7. Analysis of the Role of Transcription Factors in the Regulation of Salt Tolerance in Rice

Transcription factors play a crucial regulatory role in the response of plants to salt stress. Therefore, identifying transcription factors can help to analyse potential salt tolerance regulatory factors. We identified 83 members of 22 transcription factor families among the genes specifically responsive to salt stress. Among the salt-tolerant variety N14 and the salt-sensitive variety N6, 12 transcription factors were differentially expressed under salt stress. Of these, 7 were downregulated in both varieties, 2 were upregulated in N14 but downregulated in N6, and 3 were downregulated in N14 but upregulated in N6.

**Figure 7:**
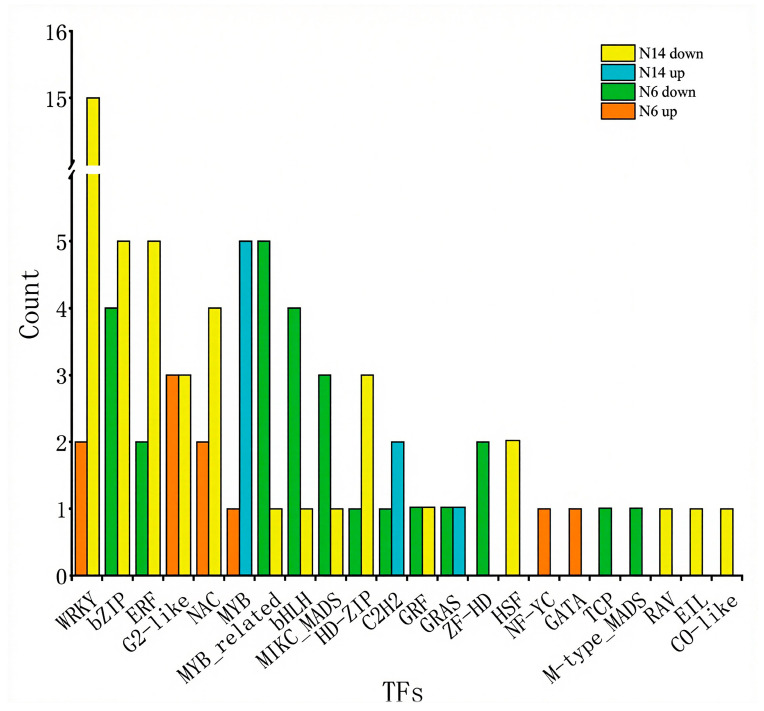
Distribution of differentially expressed transcription factors in salt-tolerant and salt-sensitive varieties under salt stress.

The transcription factor gene families primarily up-regulated in the salt-tolerant variety N14 belong mainly to the MYB gene family, while the transcription factor families primarily up-regulated in salt-sensitive varieties include G2-like, WRKY, and NAC, among others. The transcription factor families primarily down-regulated in N14 include WRKY, bZIP, and ERF, while the transcription factors primarily down-regulated in salt-sensitive varieties include bZIP, MYB-related, and bHLH, among others. This indicates that different transcription factors are involved in the salt stress response between salt-tolerant and salt-sensitive varieties, which is an important reason for the differences in salt tolerance between these varieties (Figure 7).

**Figure 8:**
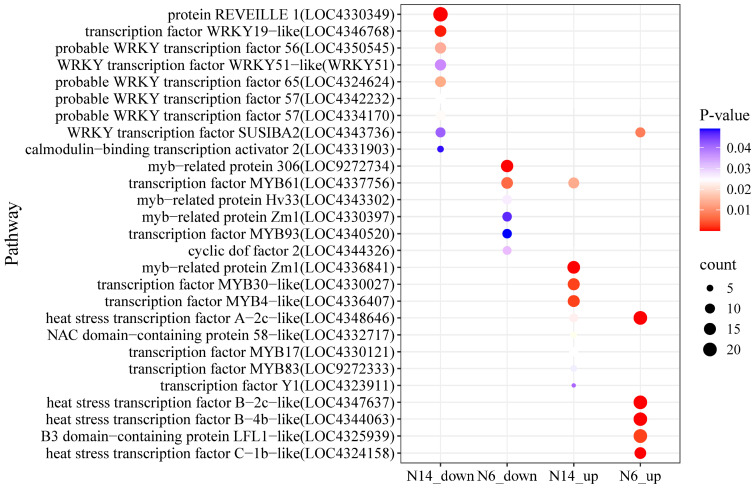
Distribution of transcription factor-regulated genes in salt-tolerant and salt-sensitive varieties under salt stress.

This study conducted enrichment analysis on differentially expressed transcription factor-regulated genes in salt-tolerant and salt-sensitive rice. In salt-tolerant varieties, genes specifically downregulated were enriched in multiple WRKY transcription factor-regulated genes (Figure 8), while in salt-sensitive varieties, genes specifically downregulated were enriched in MBY transcription factor-regulated genes; in salt-tolerant varieties, genes specifically upregulated were enriched in MYB family-regulated genes, while in salt-sensitive varieties, they were enriched in heat shock response genes.

Under salt stress conditions, transcription factor analysis of the N6 and N14 rice groups revealed that the WRKY family was significantly downregulated in the N14 group, with downregulated genes including REVEILLE 1 (LOC4330349), WRKY19-like (LOC4346768), WRKY56 (LOC4350545), and SUSIBA2 (LOC4343736); in the N6 group, the MYB family was significantly downregulated, including myb-related protein 306 (LOC9272734), MYB61 (LOC4337756), and MYB93 (LOC4340520); in the N14 group, MYB family members Zm1 (LOC4336841), MYB30-like (LOC4330027), and MYB4-like (LOC4336407) were significantly upregulated; The heat shock transcription factor family was significantly upregulated in the N6 group, including HSF B-4b-like (LOC4344063) and HSF B-2c-like (LOC4347637), and HSF A-2c-like (LOC4348646) was upregulated in both groups; Additionally, SUSIBA2 was up-regulated in N6 and down-regulated in N14, while MYB61 was down-regulated in N6 and up-regulated in N14, exhibiting opposite expression patterns.

### 3.8. Quantitative RT-PCR

Confirmation tests were carried out to evaluate the expression levels of DEGs identified from the RNA-seq analysis using qRT-PCR of randomly selected genes.

**Figure 9:**
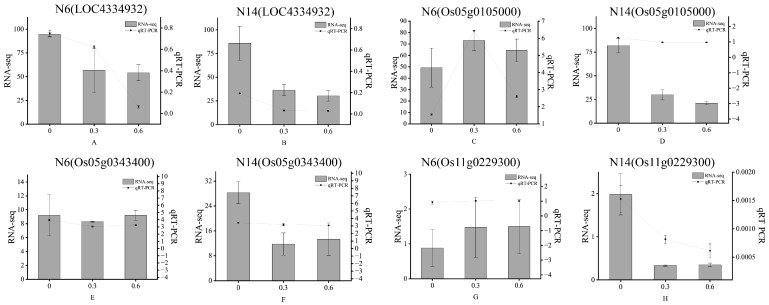
Comparison analysis of qRT-PCR and RNA-seq for 4 genes in 2 cultivars.

In order to verify the RNA-seq data, four differentially expressed genes common to the two cultivars were selected for qRT-PCR, and the results were consistent with the RNA-seq data(Figure 9).

## 4. Discussion

Rice is a crop that is extremely susceptible to salt stress, which can lead to a decrease in rice yield [17,18]. In this study, N14 showed no significant inhibition of tillering growth under salt stress, whereas N6 showed significant inhibition. This study aims to explore the molecular mechanisms underlying the response to salt stress in salt-tolerant and salt-sensitive rice varieties.

Studies have shown that high salt stress activates chlorophyll degradation enzymes, leading to a decrease in chlorophyll content [19]. Low salt stress may not have such a strong activating effect, thereby reducing chlorophyll degradation and making the leaves appear greener. Different rice varieties respond differently to salt stress. Some rice varieties can maintain better traits under high salt conditions, retain specific morphological and cellular structures, and demonstrate higher salt tolerance [20]. The results of this study indicate that high salt stress affects rice morphology, with significant differences between the two rice varieties, N6 and N14. Under increasing salt concentrations, N14 exhibits better morphological structure than N6. This is consistent with previous studies, which have shown that high salt stress severely affects rice leaf colour and overall morphology.

At the transcriptomic level, this study investigated the common response to salt stress of two varieties, N6 and N14, which exhibit significant differences in salt tolerance. The results showed that 372 DEGs were detected in N6, while 393 DEGs were detected in N14. This differs from previous studies, which found that salt-tolerant rice genotypes exhibit stronger transcriptomic reprogramming than salt-sensitive rice genotypes [14]. Therefore, it is speculated that salt-sensitive varieties may attempt to compensate for their insufficient salt tolerance mechanisms through more intense transcriptional reprogramming; in contrast, salt-tolerant varieties can maintain cellular homeostasis with a more ‘streamlined’ and efficient transcriptional regulatory network. Further comparison of variety-specific responses revealed that 17 genes exhibited consistent expression trends, with 4 genes showing downregulation and 13 genes showing upregulation. In both varieties, the number of upregulated genes exceeded that of downregulated genes. Suggesting a proactive gene expression strategy to regulate growth and stress response under saline conditions [21]. These results indicate that rice is mobilising gene expression to adapt to long-term salt stress, thereby regulating plant growth and development under salt stress conditions. Studies have shown that WRKY56 belongs to one of the largest transcription factor families in plants, the WRKY family, which plays a key role in plant growth and development, as well as responses to biotic and abiotic stresses. WRKY transcription factors regulate the expression of downstream target genes by specifically binding to cis-elements in promoters, thereby participating in plants’ adaptation to environmental stress [22]. This study found that under salt stress, the WRKY family as a whole showed a downward trend in salt-tolerant varieties. This result is highly consistent with the report of Wang, J. et al. [23] in soybeans. Therefore, it is speculated that in N14, leaves avoid ROS bursts and photosynthetic system damage by inhibiting the negative regulation of WRKY. Combining the findings that MYB61 downregulation under normal moisture conditions leads to insufficient stomatal closure and weakened transpiration flux, and the report that MYB61 directly activates SOS1 and promotes long-distance Na^+^ efflux, we speculate that MYB61 downregulation in salt-sensitive materials may limit SOS1-mediated Na^+^ transport to the aboveground parts by weakening transpiration flux, thereby exacerbating Na^+^ toxicity in the mesophyll [24,25]. Transcriptome data from this study also show that MYB61 expression significantly decreases under salt stress, suggesting that its downregulation may be a key node leading to the loss of salt tolerance. However, in salt-tolerant materials, the upregulation of MYB61 may synchronously enhance stomatal transpiration pull and SOS1-mediated Na^+^ loading, thereby transforming salt stress into a controllable ion compartmentalization process, representing a key gain node for the acquisition of salt tolerance.

In studying plant abiotic stress, salt stress has always been a widely focused-on topic in plant biology and agricultural science. The salt overly-sensitive pathway is one of the more thoroughly researched salt stress signal transduction pathways [26]. Sakamoto et al. [27] first cloned the RPR1 gene and found that it encodes an NBS-LRR-type disease resistance protein, whose expression is induced by probenazole, SA, and pathogens, and is closely related to systemic acquired resistance (SAR). Although this study did not involve salt stress, the induction pattern of RPR1 expression and its role in SAR suggest that it may be involved in a broader stress response mechanism. Wang et al. [28] identified OsRSR1, an NBS-LRR-type resistance gene, as a positive regulator of rice sheath blight resistance via activation of the GSH-AsA antioxidant system and ROS homeostasis. Given the structural similarity between OsRSR1 and RPR1, and the shared role of ROS signaling in both biotic and abiotic stress responses, it is plausible that RPR1 may also modulate salt tolerance through analogous antioxidant-mediated mechanisms. A study comparing the transcriptomes of the salt-tolerant rice variety NGY1 and its parents found that several NBS-LRR-type disease-resistant protein genes were significantly upregulated in the early stages of salt stress, suggesting that these genes may play an important role in rice salt tolerance [29]. The results suggest that the differentially expressed gene OsRSR1 (Os11g0229300) in N6 and N14 may enhance rice salt tolerance by mediating NBS-LRR-type disease-resistant proteins. Research has shown that LOC_Os10g32980 (OsCESA7) is upregulated in the roots of the stress-tolerant variety (NIL-23) under phosphorus deficiency stress, suggesting its role in the response to abiotic stress [30]. This gene shows a significant downregulation in N6 under salt stress but is markedly upregulated in N14. Therefore, it is speculated that this gene participates in the molecular regulatory mechanism of rice salt stress tolerance by regulating the dynamic synthesis of secondary cell wall cellulose. In this study, LOC_Os06g45140 (OsbZIP52) showed a downregulation trend in both N6 and N14, consistent with reports from [31] and [32]: this gene is significantly induced by low temperatures and has been confirmed to be a negative regulator of cold-drought stress. OsbZIP52 suppresses the expression of osmotic protection genes such as OsLEA3, OsTPP1, and Rab25 by binding to the G-box element. Its downregulation helps to release this suppression, indirectly activating downstream stress-tolerance pathways. Therefore, this expression change may be an active transcriptional regulation strategy implemented by plants to enhance salt tolerance. Previous studies have shown that LOC_Os05g44340 (HSP101) exhibits significantly upregulated expression following high-temperature stress in the evaluation of heat tolerance in early-stage seedlings of indica rice varieties [33]. This study found that under salt stress conditions, LOC_Os05g44340 (HSP101) exhibited upregulation in both N6 and N14, suggesting that this gene not only participates in heat stress responses but may also play a conserved cellular protective role in salt stress responses.

## 5. Conclusions

**Figure 10:**
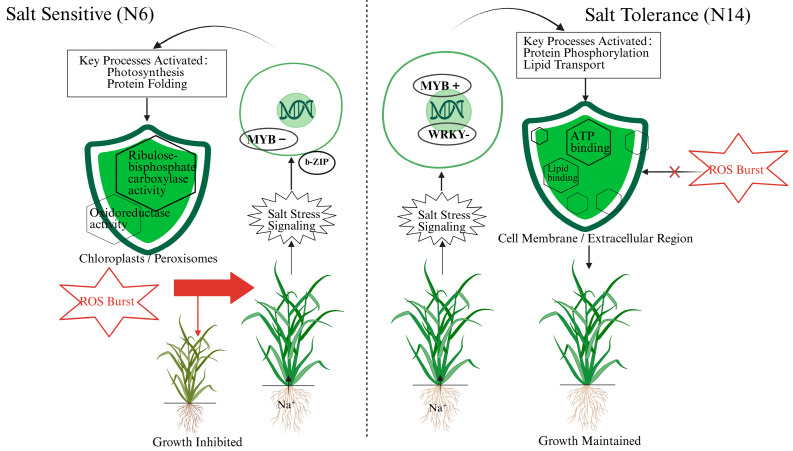
Contrasting molecular response pathways to salt stress in salt-sensitive (N6) and salt-tolerant (N14) rice varieties.

To intuitively summarize the distinctly different salt stress response mechanisms between the salt-tolerant variety N14 and the salt-sensitive variety N6, we constructed a summary model (Figure 10).

As shown in the figure, when faced with salt stress, the signaling pathway activated in the salt-sensitive variety N6 primarily involves the MYB family (significantly downregulated) and the b-ZIP transcription factor family. This signaling pathway activates biological processes related to photosynthesis and protein folding. According to GO analysis, these activities are primarily localized in chloroplasts and peroxisomes and involve molecular functions such as ribulose-bisphosphate carboxylase activity and oxidoreductase activity. However, this response pathway is insufficient to cope with the stress, leading to a reactive oxygen species ROS burst, which ultimately causes leaf yellowing and significant growth inhibition. In contrast, the salt-tolerant variety N14 initiates a more robust defense strategy. Its response involves a different set of transcription factors, notably the WRKY family (significantly downregulated) and the MYB family (upregulated). This signal transduction activates key biological processes such as protein phosphorylation and lipid transport. These processes primarily occur in the membrane and extracellular regions and rely on molecular functions such as ATP binding and lipid binding. This response, centered on membrane integrity and signaling (consistent with activated pathways like Plant-pathogen interaction), enables N14 to effectively avoid or neutralize the ROS burst, thereby maintaining growth homeostasis under salt stress and exhibiting stronger tolerance.

## Figures and Tables

**Table 1:** Preparation of mixtures.

Reaction Component	Volume (μL)
2× PerfectStart Green qPCR SuperMix	10
Primer F (10 μM)	0.4
Primer R (10 μM)	0.4
Universal Passive Reference Dye (50×)	0.4
Template (cDNA)	3
Nuclease-free Water	5.8
Total volume	20

**Table 2:** PCR cycle conditions.

Thermal Cycler	Times and Temperatures		
	Initial Steps	Each of 40 Cycles	
		Melt	Anneal and Extend
ABI 7500 Fluorescence Quantitative PCR Instrument	HOLD	CYCLE	
	30 s, 94 °C	5 s, 94 °C	30 s, 60 °C

**Table 3:** Statistics of transcriptome sequencing data.

Sample	Raw Reads	Raw Bases	Clean Reads	Clean Bases	Q20 (%)	Q30 (%)	Uniquely Mapped
N_601_	39,464,308	5,959,110,508	38,990,916	5,882,023,917	98.21	94.71	98.8
N_602_	51,013,046	7,702,969,946	50,372,526	7,599,293,771	98.2	94.76	98.74
N_603_	40,409,308	6,101,805,508	39,937,812	6,023,959,679	98.28	94.91	98.83
N_631_	45,783,182	6,913,260,482	45,176,752	6,812,770,329	98.11	94.51	98.68
N_632_	43,296,732	6,537,806,532	42,745,204	6,449,522,466	98.21	94.72	98.73
N_633_	47,153,418	7,120,166,118	46,517,786	7,018,162,788	98.07	94.41	98.65
N_661_	52,436,384	7,917,893,984	51,786,504	7,812,145,804	98.16	94.58	98.76
N_662_	53,781,444	8,120,998,044	53,054,258	8,004,198,957	98.03	94.28	98.65
N_663_	53,979,786	8,150,947,686	52,672,372	7,922,741,502	96.81	94.39	97.58
N_1401_	52,296,726	7,896,805,626	51,640,960	7,787,514,102	98.22	94.76	98.75
N_1402_	52,260,026	7,891,263,926	51,638,610	7,789,508,812	98.16	94.56	98.81
N_1403_	63,166,482	9,538,138,782	62,371,706	9,407,697,491	98.18	94.69	98.74
N_1431_	54,486,464	8,227,456,064	53,806,856	8,113,932,261	98.19	94.68	98.75
N_1432_	51,756,590	7,815,245,090	51,110,208	7,708,457,113	98.2	94.72	98.75
N_1433_	56,413,064	8,518,372,664	55,703,962	8,400,334,667	98.22	94.8	98.74
N_1461_	44,061,956	6,653,355,356	43,471,502	6,556,346,176	98.13	94.57	98.66
N_1462_	50,432,982	7,615,380,282	49,797,504	7,510,443,677	98.23	94.81	98.74
N_1463_	46,193,074	6,975,154,174	45,552,594	6,871,893,728	98.03	94.35	98.61

Note: In this table, N_601_, N_602,_ and N_603_ mean three replicates of the control group of N6 that did not undergo salt stress treatment; N_631_, N_632,_ and N_633_ mean three replicates of N6 that underwent 0.3% salt stress treatment; N_661_, N_662,_ and N_663_ mean three replicates of N6 that underwent 0.6% salt stress treatment. N14 is the same as N6.

**Table 4:** Statistics on genes differentially expressed under two salt stress conditions.

Combination	Up-Regulated	Down-Regulated	ALL DEGs
N60-N63-vs-N60-N66	252	190	442
N140-N143-vs-N140-N146	167	354	521

## Data Availability

The data that support the findings of this study are available on request from the corresponding author. From: Department of Agriculture and Resources and Environment, Tianjin Agricultural University, Tianjin, China.

## Supplementary Materials

The following supporting information can be downloaded at: https://www.mdpi.com/article/10.3390/cimb47100832/s1.
